# SUMOylation Is Required for Optimal TRAF3 Signaling Capacity

**DOI:** 10.1371/journal.pone.0080470

**Published:** 2013-11-18

**Authors:** Sophia Miliara, Kalliopi K. Gkouskou, Tyson V. Sharp, Aristides G. Eliopoulos

**Affiliations:** 1 Molecular and Cellular Biology Laboratory, University of Crete School of Medicine, Heraklion, Greece; 2 Laboratory of Cancer Biology, Institute of Molecular Biology and Biotechnology, Foundation of Research and Technology Hellas, Heraklion, Greece; 3 Barts Cancer Institute, Queen Mary University of London, London, United Kingdom; Loyola University Chicago, Stritch School of Medicine, United States of America

## Abstract

TNF receptor–associated factors (TRAFs) are multifunctional adaptor proteins involved in temporal and spatial coordination of signals necessary for normal immune function. Here, we report that TRAF3, a TRAF family member with a key role in Toll-like and TNF family receptor signaling and suppressor of lymphomagenesis, is post-translationally modified by the small ubiquitin-related modifier (SUMO). Through yeast two-hybrid and co-immunoprecipitation assays we have identified Ubc9, the SUMO conjugating enzyme, as a novel TRAF3-interacting protein. We show that Ubc9-dependent SUMOylation of TRAF3 modulates optimal association with the CD40 receptor, thereby influencing TRAF3 degradation and non-canonical NF-κB activation upon CD40 triggering. Collectively, our findings describe a novel post-translational modification of a TRAF family member and reveal a link between SUMOylation and TRAF-mediated signal transduction.

## Introduction

TNF receptor–associated factors (TRAFs) are multifunctional intracellular proteins which participate in the recruitment and activation of a plethora of protein kinases involved in immune and death receptor signaling. Among the seven known mammalian TRAFs, TRAF2, TRAF3 and TRAF6 have been extensively studied as their ablation severely impairs Toll-like and TNF family receptor signal transduction.

TRAF3 was originally identified as a molecule that binds the cytoplasmic domains of the TNF receptor family member CD40 and the oncogenic Epstein-Barr virus-encoded latent membrane protein 1 (LMP1) [1], [2], [3](reviewed in [4]). Subsequently, TRAF3 was shown to interact with the cytoplasmic tails of other TNF receptor family members, such as lymphotoxinβ-receptor (LTβR), OX40, CD27, CD30, Herpes virus entry mediator and receptor activator of NF-κB [5], [6], [7] and more recently to participate in Toll-like receptor signaling and to regulate the production of pro-inflammatory cytokines and type I interferons in response to a variety of pathogen-associated molecular patterns [8], [9]. The participation of TRAF3 in signal transduction from diverse receptors reflects its major immunological role in host defense. Indeed, TRAF3 deficiency has been linked to lymphoid defects in the mouse and increased susceptibility to herpes simplex virus-1 (HSV-1)-induced encephalitis (HSE) in humans [10], [11].

The impact of TRAF3 deregulation extends to the pathogenesis of malignancy. Inactivating mutations and loss of heterozygosity in the TRAF3 locus have been reported in a significant proportion of myeloma patients, suggesting that TRAF3 may function as a tumor suppressor [12], [13]. In line with this notion, we and others have previously demonstrated that TRAF3 mediates the anti-proliferative effects of CD40 and LTβR in transformed cell lines [14], [15] and that overexpressed TRAF3 inhibits the growth of carcinoma [14] and myeloma cells [13].

The role of TRAF3 in disease pathogenesis has partly been attributed to the engagement of the ‘alternative’ NF-κB pathway which involves the processing of p100 NF-κB2 to p52. In the absence of stimulus, TRAF3 is required for the formation of a cytoplasmic multi-protein complex containing TRAF2, NF-κB inducing kinase (NIK) and the cellular inhibitors of apoptosis (cIAP1/2). In this complex, the ubiquitin ligase activity of cIAPs is channeled towards NIK leading to its degradation [16], [17]. Indeed, ablation of TRAF3 in the mouse or reduced TRAF3 levels in myeloma [12], [13] and HSE patients [18] elevate NIK expression resulting in constitutive activation of the NF-κB2 pathway and accumulation of transcriptionally active p52. Deregulated NF-κB2 signaling accounts for the postnatal lethality associated with TRAF3 ablation [9].

Transient activation of p100 processing occurs in response to stimulation of TRAF3-interacting receptors, such as CD40 [19]. Ligation of CD40 in B cells results in rapid recruitment of a multiprotein complex containing TRAF2, TRAF3 and cIAP1/2 to the receptor. TRAF2 serves not only as adaptor protein but also as E3 ubiquitin ligase which functions together with the E2 ubiquitin-conjugating enzyme Ubc13 to catalyze the addition of lysine 63 (K63)–linked polyubiquitin chains to cIAP1/2. This, in turn, activates the ubiquitin ligase activity of cIAP1/2 toward TRAF3, catalyzing degradative K48–linked polyubiquitination. The ensuing degradation of TRAF3 results in NIK accumulation and activation through autophosphorylation [20]. Whereas TRAF3 negatively regulates alternative NF-κB2 signaling, it is required for the activation of IRF3 and the interferon (IFN) response, an effect attributed to K63 rather than K48-mediated Ub linkages [20].

The function of TRAFs in signal transduction exceeds, therefore, their role as molecular bridges between receptors and intracellular signaling components and includes active regulation of kinase activation through inducible ubiquitination. In this study using yeast two-hybrid cloning and co-immunoprecipitation assays we have identified Ubc9 as a novel TRAF3-interacting protein and demonstrated that TRAF3 is subject to an additional post-translational modification, namely SUMOylation. Furthermore, we show that the TRAF3:Ubc9 association affects the ability of TRAF3 to bind CD40 and signal on the non-canonical CD40-NF-κB2 axis.

## Materials and Methods

### Yeast two-hybrid screening

TRAF3 cloned into the GAL4 DNA-binding vector pAS1 was kindly provided by Professor George Mosialos (Aristotle University of Thessaloniki, Greece) and used as bait in a two-hybrid screening of HeLa cDNA library according to the Matchmaker Two-hybrid System Protocol (Clontech). Positive clones were recovered and sequenced. Interactions with Ubc9 were confirmed by transfection into the *S. cerevisiae* strain PJ69-4A and examination of growth in media lacking tryptophan and leucine (to select for the introduced plasmids) and also lacking either adenine (SD-leu-trp-ade), histidine but supplemented with 10 mM 3-aminotriazole (SD-leu-trp-his + 10 mM 3-AT) or containing X-gal (SD-leu-trp + 40 µg/ml X-gal) to assay galactosidase reporter gene expression.

### Cell culture and transfections

EJ cells [28] were maintained in RPMI medium supplemented with 10% fetal bovine serum. 293T, a variant of the human embryonic kidney cell line 293 (ATCC, CRL3216) and HeLa cells stably expressing His-SUMO-1 and His-SUMO-2 (kindly provided by Prof. Ron Hay, University of Dundee, UK, [21]), were maintained in Dulbecco’s modified Eagle’s Medium (DMEM, Gibco) supplemented with 10% fetal calf serum in an atmosphere of 5% CO2, at 37°C. Transient transfections were performed using Lipofectamine (Invitrogen) according to the manufacturer’s protocol. Isolation of primary splenocytes was performed as previously described [19].

### Plasmids

SUMO expression vectors were gift from Prof. Ron Hay. Human wild type Ubc9 was PCR amplified from a HeLa cDNA library using Expand High Fidelity PCR System (Roche, Germany) and the following primers: sense Bam-UBC-FORW 5′-CTTTGAACGGATCCGGGATCGCCCTC- 3′ and antisense Eco-UBC-REV 5′- CACAAGGTGAATTCTTATGAGGGCGCAAAC- 3′ (MWG, Germany). The PCR amplification conditions were as follows: 94°C for 3 minutes followed by 29 cycles of 94 °C for 30 seconds, 64°C for 40 seconds and 72°C for 45 seconds and a final extension step of 72°C for 5 minutes. A mutated Ubc9 carrying a substitution of Cys^93^ in the active site of the enzyme with Ala which is unable to form SUMO conjugates was provided by Dr Y.Y. Mo [22]. TRAF3 deletion mutants corresponding to aa 1-266 (Mutant 1), aa 1-376 (Mutant 2), aa 267-568 (mutant 3) and aa 346-568 (mutant 4) were generated by PCR using the following primers:

Mutant 1F: 5′-GGAATTCAGTAAAAAGATGGACTCTCCTG-3′,

Mutant 1R: 5′-CGACTCGAGTCACTCCTTCAGCAGG-3′,

Mutant 2F: 5′-GGAATTCAGTAAAAAGATGGACTCTCCTG-3′,

Mutant 2R: 5′-ACTCGAGCAGGCCTCAGTTCCGAGC-3′

Mutant 3F: 5′-GGAATTCAGCAACTCGCTCGAAAAGAAG-3′,

Mutant 3R: 5′-CCTCGAGTCAGGGATCGGGCAG-3′,

Mutant 4F: 5′-GGAGGAATTCGACAGCATGAAGAGCA-3′,

Mutant 4R: 5′ CCTCGAGTCAGGGATCGGGCAG 3′

PCR conditions were as follows: for mutant 1 and 4, 94°C for 2 minutes followed by 30 cycles of 94 °C for 30 seconds, 61°C for 40 seconds and 72°C for 50 seconds and then extended by 72°C for 10 minutes; for mutants 2 and 3, 94°C for 2 minutes followed by 30 cycles of 94 °C for 30 seconds, 61°C for 40 seconds and 72°C for 67 seconds and then extended by 72°C for 10 minutes. The TRAF3 mutants were cloned as EcoRI/XhoI fragments into a modified pcDNA3.1 (Invitrogen, CA, USA) vector containing a FLAG-tag and sequenced.

### Preparation of protein extracts

Depending on the experimental setting, cells were lysed in different buffers. For native immunoprecipitations, cells were lysed in RIPA buffer (25 mM Tris-HCI pH 7.2, 50 mM NaCI, 0.5% NP40, 0.5% sodium deoxycholate) supplemented with 1% SDS and 25 mM N-Ethylmaleimide or iodoacetamide (Sigma). Alternatively, cells were lysed in Guanidinium-HCI to allow purification on Ni-NTA columns [21].

### Co-Immunoprecipitation Studies

Cell lysates were cleared and incubated with anti-TRAF3 antibody (1 µg/mg lysate) and G-Sepharose Beads (20 µl) for 12 hours at 4°C on a rotor. The beads were washed extensively in Lysis Buffer A [23] and boiled with 30 µl Protein Loading Buffer [50 mM Tris pH 6.8, 4% SDS (w/v), 10% glycerol, 5% mercaptoethanol, 0.01% bromophenol blue (w/v)].

### Western Analysis

Samples were analyzed by SDS-PAGE and transferred onto polyvinylidenedifluoride membrane (0.45 µM; Millipore). The membranes were incubated with primary antibodies overnight at 4°C: TRAF3 (C20), TRAF2 (C20), IκBα (C21), RIP1 (C20) were purchased from Santa-Cruz Biotechnology), anti-myc tag 9E10, anti-FLAG M2 and β-actin antibodies were from Sigma and NF-κB2 antibody from Cell Signaling Technology. Anti-SUMO-1 and SUMO-2 antibodies were kindly provided by Professor Ron Hay, University of Dundee, UK. Following 1 h incubation with the appropriate secondary antibody, membranes were subjected to enhanced chemiluminescence analysis.

### GST Pull-Down Assays

Isolation and purification of GST or GST-Ubc9 fusion protein were performed as previously described [23]. For pull-down assays, 500 µg total protein isolated from HEK293 cells was mixed with beads carrying GST or GST-Ubc9 and incubated for 2 h at 4°C. After three washes with PBS, proteins were solubilized in 1 × SDS protein sample buffer and separated in 8% SDS polyacrylamide gels.

## Results

### Isolation of Ubc9 as a TRAF3-interacting protein in yeast

To increase our understanding of the function of TRAF3, we performed yeast two-hybrid interaction assays to search for proteins that directly associate with TRAF3. An expression vector in which full-length TRAF3 was fused to the Gal4 DNA binding domain was used as bait to screen a HeLa cDNA library [24]. From approximately 10 million transformants, 10 independent positive clones were obtained. One of them encoded the full-length Ubc9, a protein associated with the process of SUMOylation.

**Figure 1:**
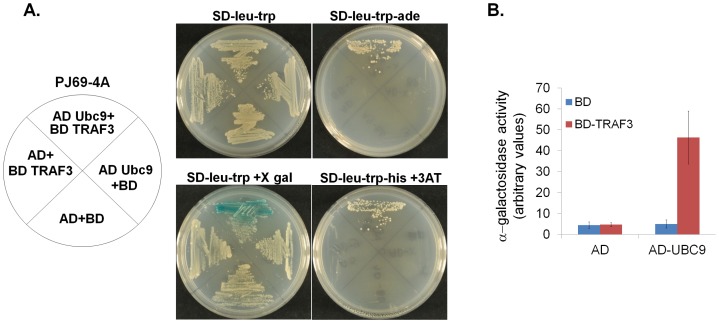
TRAF3 interacts with Ubc9 in a yeast 2-hybrid assay. (**A**) *S. cerevisiae* strain PJ69-4A was transformed with plasmids expressing the GAL4 activation domain (AD) fused to Ubc9, the GAL4 binding domain (BD) fused to TRAF3 or with control empty vectors in all possible combinations. Cells were grown on standard medium (SD-leu-trp) in the presence or absence of X gal (SD-leu-trp+X gal), medium lacking adenine (SD-leu-trp-ade) to detect expression of the *GAL-ADE2* reporter gene or medium lacking histidine (SD-leu-trp-his + 5 mM 3-AT) to detect expression of the *GAL-HIS3* reporter gene. (**B**) Quantification of galactosidase reporter activity in yeast transformed with the vectors described in (A). Results shown represent the mean values of galactosidase activity (±SD) relative to controls from 4 independent experiments.

To verify this interaction, *S. cerevisiae* strain PJ69-4A was transformed with plasmids expressing the GAL4 activation domain (AD) fused to Ubc9, the GAL4 binding domain (BD) fused to TRAF3 or with control empty vectors in all possible combinations. Cells were grown in standard medium (SD-leu-trp) in the presence or absence of X gal, in medium lacking adenine (SD-leu-trp-ade) to detect expression of the *GAL-ADE2* reporter gene or in medium lacking histidine (SD-leu-trp-his + 3-AT) to detect expression of the *GAL-HIS3* reporter gene. The growth patterns of the transformed cells and the expression levels of β-galactosidase activity confirmed that TRAF3 and Ubc9 interact in yeast (Fig. 1).

### TRAF3 associates with Ubc9 through its ring finger TRAF-N domain

**Figure 2:**
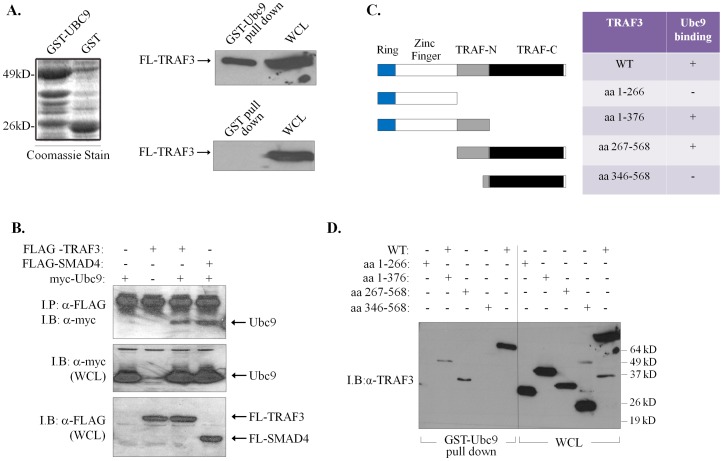
TRAF3interacts with Ubc9 through its ring finger TRAF-N domain. (**A**) GST-Ubc9 produced in bacteria as fusion with GST interacts with FLAG-tagged TRAF3 expressed in HEK 293 cells. GST or GST-Ubc9 was incubated with protein lysates isolated from FLAG-TRAF3 transfected HEK293 cells. Pulled-down proteins were solubilized in SDS protein sample buffer, separated on 8% SDS polyacrylamide gels and immunoblotted for TRAF3. Coomassie blue staining was used to confirm equal utilization of GST and GST-Ubc9 proteins in the pull-down assays. (**B**) Ubc9 and TRAF3 interact *in vivo*. FLAG-tagged TRAF3 was co-expressed with myc-tagged Ubc9 in HEK293 cells. Ubc9 was detected in anti-FLAG immunoprecipitates. As positive control [25], FLAG-tagged SMAD4 was used to monitor interactions with Ubc9. (**C &D**) Interaction of TRAF3 deletion mutants with Ubc9 in an *in vitro* GST pull-down assay. 293T cells were transiently transfected with the FLAG-tagged TRAF3 deletion mutants shown in *C*. Thirty six hours later, the extracts were incubated with purified GST-Ubc9 bound to glutathione sepharose beads. Interacting proteins were fractionated by SDS-PAGE and immunoblotted with anti-FLAG mAb (lanes 1-5; *D*). Whole cell lysates (30 µg) were analyzed in parallel to monitor the motility of the TRAF3 deletion mutant proteins (lanes 6-10). *I.P*.; immunoprecipitation. *I.B*.; immunoblot. *WCL*; Whole cell lysates

To further validate the interaction between Ubc9 and TRAF3, a fusion of GST with Ubc9 was examined for its ability to interact with FLAG-tagged TRAF3 expressed in human embryonic kidney (HEK) 293 cells. As shown in Figure 2A, GST-Ubc9 strongly associated with TRAF3 whereas control GST did not. This interaction was further analyzed in mammalian cell co-immunoprecipitation assays. The full-length Ubc9 construct containing an N-terminal Myc epitope tag (myc-Ubc9) was transiently co-expressed in HEK 293 cells with FLAG-tagged TRAF3 or, as positive control, SMAD4 [25]. Cell lysates were immunoprecipitated using a monoclonal antibody against the FLAG epitope, and co-precipitating Ubc9 was detected by immunoblotting with anti-Myc polyclonal antibody. As shown in Figure 2B, FLAG-TRAF3 interacted with Myc-Ubc9.

TRAF3 contains a N-terminal ring finger domain followed by several zinc fingers [3]. The C-terminal half of TRAF3 comprises the TRAF domain, which is conserved among all members of the TRAF family and can be further subdivided into the TRAF-N and TRAF-C regions [1]. To identify the TRAF3 domain which contributes to Ubc9 binding, various FLAG-tagged TRAF3 deletion mutants were generated (Fig. 2C) and assayed for association with GST-Ubc9. The results of the GST pull-down assays demonstrated that the TRAF-N domain of TRAF3 is predominantly responsible for binding to Ubc9 (Fig. 2D). However, binding was reduced compared to wild-type TRAF3 indicating conformational effects on effective interaction with Ubc9.

### Ubc9 is required for SUMO modification of TRAF3

Similar to ubiquitin, SUMO is covalently conjugated to a substrate protein lysine through a reaction that utilizes an activating E1 enzyme, a conjugating E2 enzyme and an E3 ligase. Unlike the ubiquitination pathway where each E2 has a specific set of target proteins, SUMO conjugation is mediated exclusively by Ubc9. The association of TRAF3 with Ubc9 (Fig. 1 and 2) led us to postulate that TRAF3 is a SUMO substrate. In vertebrates, two SUMO protein subfamilies, SUMO-1 and SUMO-2/3 have mostly been studied. SUMO-2 and SUMO-3 are commonly referred to as SUMO-2/3 due to 98% sequence similarity and the lack, to date, of distinguishable functional differences.

**Figure 3:**
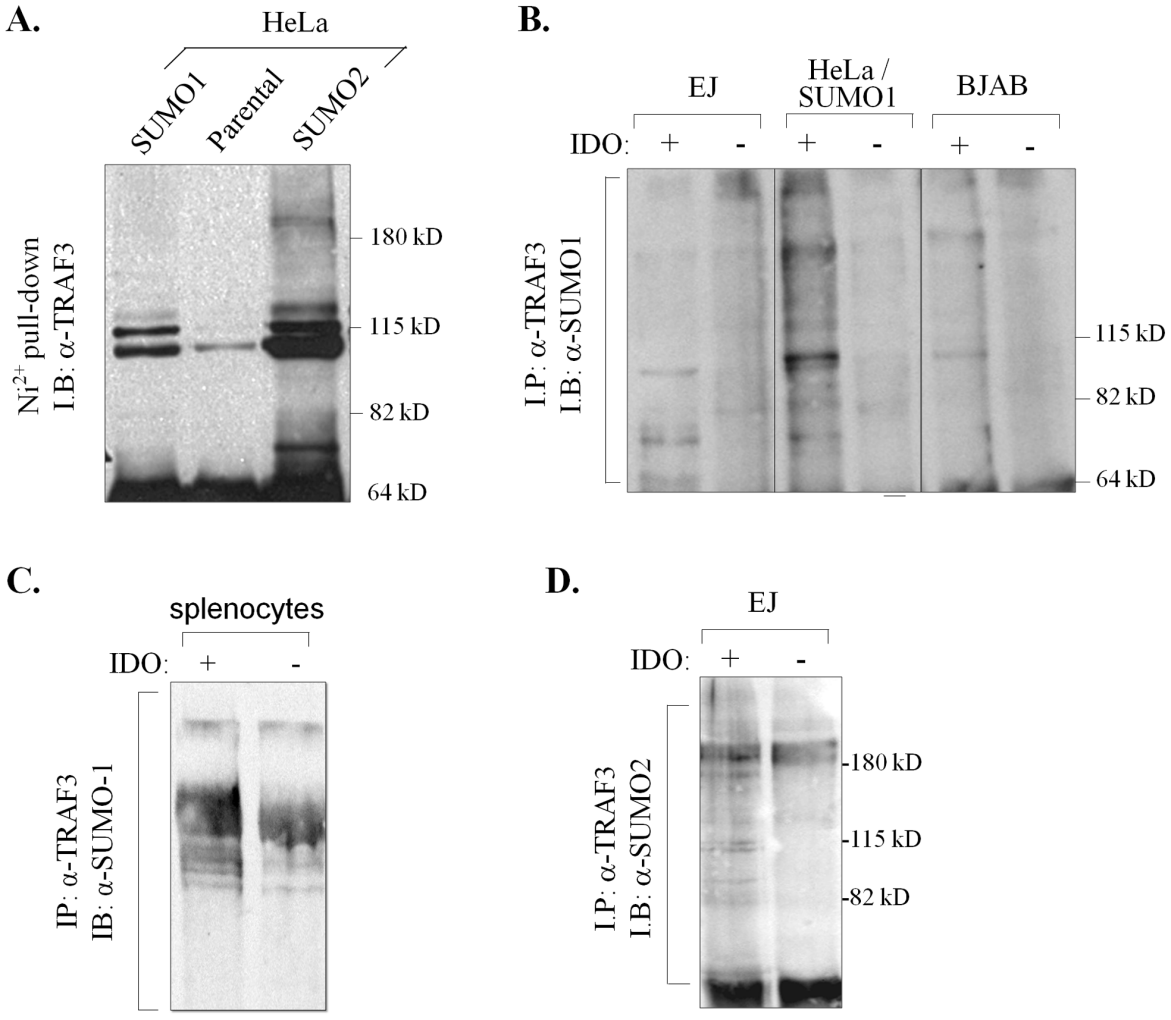
TRAF3 is post-translationally modified by SUMO. (**A**) HeLa cells stably expressing His-tagged SUMO-1 or SUMO-2 and parental cells were lysed in a protein-denaturing buffer and lysates were subjected to enrichment of SUMOylated proteins on nickel-nitrilotriacetic acid (Ni-NTA) columns. Eluates were immunoblotted with a TRAF3 polyclonal antibody. (**B & C**) SUMO-1 modification of TRAF3. Protein lysates were obtained from EJ bladder carcinoma, BJAB lymphoma and HeLa cervical carcinoma cells stably transfected with SUMO-1 (B) or mouse splenocytes (C) in the presence or absence of iodocetamide (IDO) and immunoprecipitated (*I.P*) with anti-TRAF3 C20 antibody. The SUMO-1 conjugates were detected by anti-SUMO-1 specific antibody. (**D**) SUMO-2/3 modification of TRAF3. Protein lysates were obtained from EJ cells in the presence orabsence of IDA and TRAF3 immunoprecipitates (*I.P*) were immunoblotted (*I.B*) with an anti-SUMO-2/3 specific antibody.

HeLa cells stably expressing His-tagged SUMO-1 or SUMO-2 and parental cells were lysed in denaturing conditions to protect SUMO conjugation from the action of SUMO-deconjugating enzymes and lysates were subjected to enrichment of SUMOylated proteins on nickel-nitrilotriacetic acid (Ni-NTA) columns. Eluates were immunoblotted with α-TRAF3 polyclonal antibody. The results showed immunoreactive bands with increased molecular mass corresponding to endogenous TRAF3-SUMO conjugates in eluates from both His-SUMO-1 and SUMO-2-expressing HeLa clones (Fig. 3A).

To further validate this finding, lysates from EJ bladder carcinoma, BJAB lymphoma and HeLa/His-SUMO-1 cervical carcinoma cells were immunoprecipitated with anti-TRAF3 and precipitates were immunoblotted with anti-SUMO-1 antibody. Using normal lysis conditions we were unable to detect TRAF3-SUMO-1 conjugates (Fig. 3B, lanes 2, 4 and 6). As SUMOylation is readily reversible, we included the cysteine peptidase inhibitor iodoacetamide (IDO) in the lysis buffer which inhibits SUMO-deconjugating enzymes thus facilitating detection of SUMOylated proteins. Addition of IDO allowed detection of higher molecular weight bands representing SUMO-1-modified TRAF3 (Fig. 3B, lanes 1, 3 and 5). Similar results were obtained by analyzing lysates from mouse splenocytes, suggesting that SUMO-1 modification of TRAF3 is not a phenomenon restricted to transformed cells (Fig. 3C). TRAF3 was also found to be substrate for SUMO-2/3 in EJ cells (Fig. 3D).

**Figure 4:**
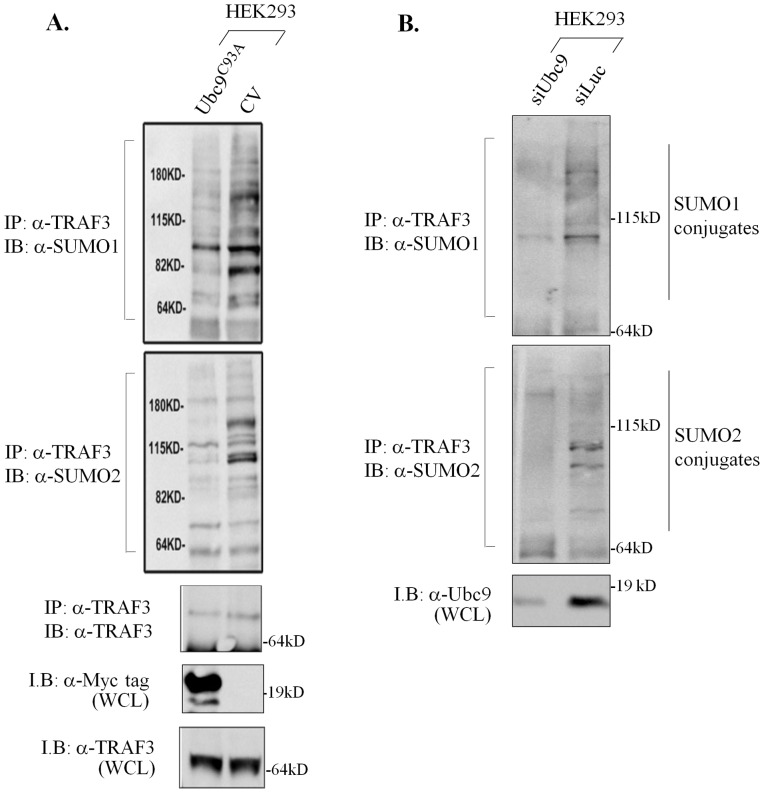
TRAF3 SUMOylation is Ubc9-dependent. (**A**) Over-expression of “dominant-negative” Ubc9^C93A^ which is unable to catalyze formation of SUMO conjugates reduces endogenous TRAF3 SUMOylation. HEK293 cells were transfected with Ubc9^C93A^ and lysates were obtained using an IDO-containing lysis buffer. Anti-TRAF3 immunoprecipitates (*I.P*) were immunoblotted (*I.B*) with either anti-SUMO-1 or anti-SUMO-2/3 antibodies, as indicated. Lysates were also immunoblotted with anti-MYC tag antibody to confirm expression of Ubc9^C93A^ or anti-TRAF3 to detect the levels of immunoprecipitated endogenous TRAF3. *CV*; control vector. (**B**) Knock-down of Ubc9 diminishes TRAF3 SUMOylation. HEK 293 cells were transfected with Ubc9 siRNA or an unrelated siRNA targeting *luciferase* (Luc) prior to lysis. Anti-TRAF3 immunoprecipitates were then immunoblotted with either anti-SUMO-1 or anti-SUMO-2 antibodies, as indicated. Results are representative of at least 4 independent experiments for (A) and (B).

To validate the link between Ubc9 and TRAF3 SUMOylation, we targeted Ubc9 in HEK 293 cells by either over-expressing a ‘dominant negative’ mutant or by RNA interference (RNAi). The mutated Ubc9 used in these experiments, Ubc9^C93A^, carries a substitution of Cys^93^ with Ala in the active site of the enzyme which renders it unable to catalyze formation of SUMO conjugates [22]. Endogenous TRAF3 was immunoprecipitated from cell lysates using an anti-TRAF3 polyclonal antibody and immunoblotted with anti-SUMO-1 or anti-SUMO-2/3 antibodies. As shown in Figure 4A, immunoreactive bands corresponding to TRAF3-SUMO conjugates were detected in control-transfected HEK 293 cell immunoprecipitates but their intensity was reduced in the presence of dominant negative Ubc9. Similarly, the RNAi-mediated knock-down of Ubc9 in EJ cells also led to significant reduction in the levels of SUMOylated TRAF3 (Fig. 4B). These data underscore the functional significance of the TRAF3:Ubc9 interaction in the SUMO modification of TRAF3.

### Ubc9 is required for efficient TRAF3 degradation following CD40 receptor occupancy

**Figure 5:**
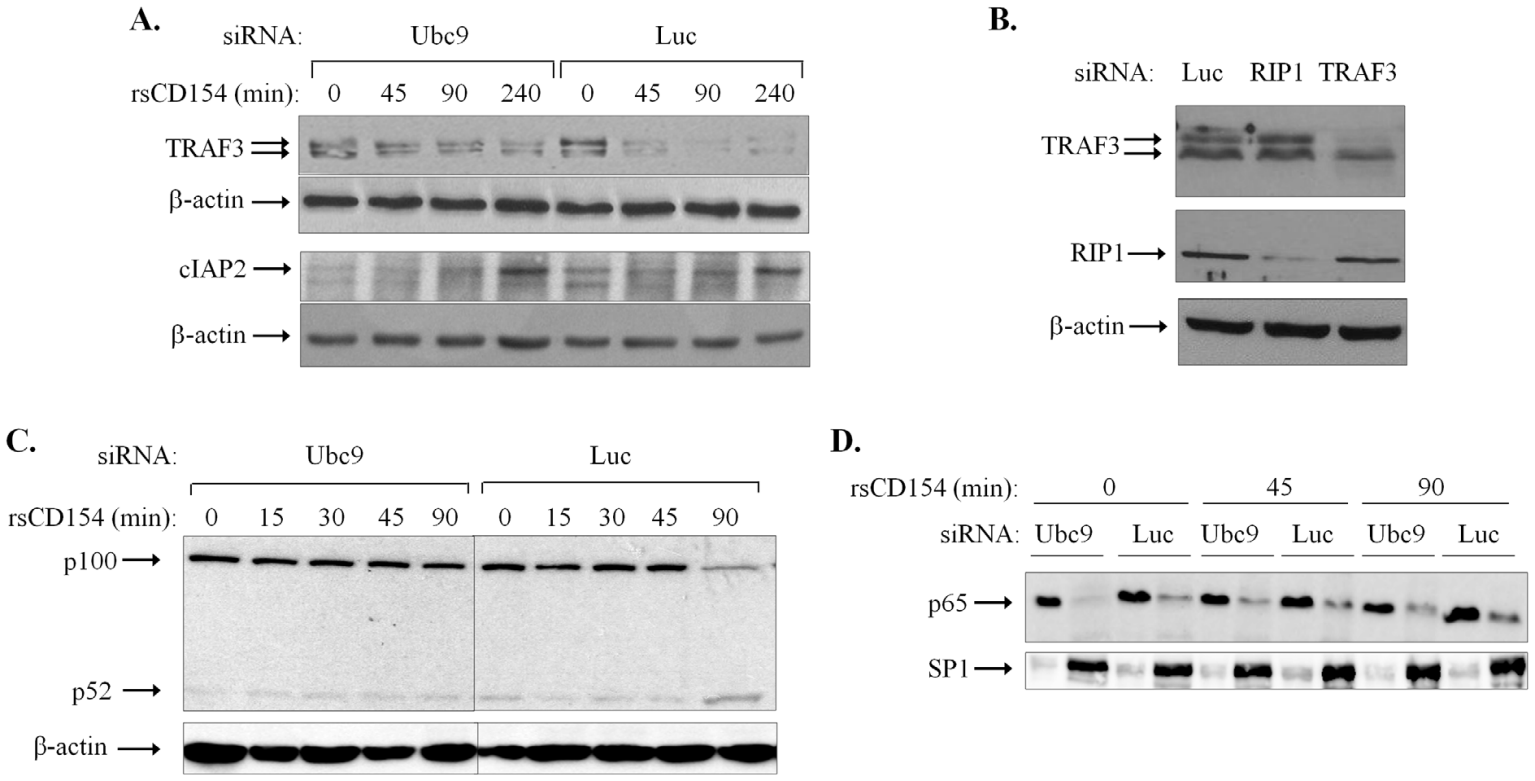
SUMOylation affects TRAF3 degradation and non-canonical NF-κB2 signaling in CD40-stimulated cells. **(A)** Knock-down of Ubc9 diminishes CD40 ligand (CD154) induced TRAF3 degradation. EJ cells were transfected with Ubc9 siRNA or an unrelated siRNA targeting *luciferase* (Luc) prior to stimulation with 0,5 µg/ml recombinant CD154 and lysis. Lysates were immunoblotted with anti-TRAF3, anti-cIAP2 or anti-β-actin antibodies. Results are representative of at least 5 independent experiments. (**B**) Validation of siRNA targeting TRAF3. EJ bladder carcinoma cells were transfected with siRNAs against TRAF3, RIP1 or the unrelated *luciferase* and knock-down efficacy and specificity were determined by immunoblotting cell lysates with anti-TRAF3, RIP1 or β-actin antibodies. (**C**) Lysates from Ubc9 knocked-down cells were immunoblotted with anti-NF-κB2 mAb recognizing both the full-length p100 and processed p52 form of NF-κB2 or with β-actin as loading control. Results are representative of 3 independent experiments. (**D**) Nuclear and cytoplasmic protein extracts were prepared from control and Ubc9 siRNA-transfected EJ cells before and after stimulation with 0,5 µg/ml CD40L and analyzed for p65/RelA expression by immunoblot. The transcription factor Sp1 was used as a marker for the purity of the nuclear cell extract preparation. Results are representative of 3 independent experiments.

TRAF3 directly interacts with and participates in CD40 signaling. In particular, CD40 activation in B cells results in cIAP-mediated ubiquitination and degradation of TRAF3 [26], [27] which is required for NIK stabilization and non-canonical NF-κB2 signal activation [16], [17]. We have examined the impact of Ubc9 ablation on CD40-mediated TRAF3 degradation in EJ cells, a cell line that was fundamental in the discovery of CD40 [28] and functionally responds to CD40 receptor occupancy [29]. As shown in Figure 5A, EJ cells transfected with control siRNA showed progressive loss of TRAF3 upon CD40 stimulation whereas the knock-down of endogenous Ubc9 attenuated this effect. As control, the levels of cIAP2, a known CD40 ligand (CD154)-inducible gene [30], [31] did not differ between control and Ubc9 siRNA-transfected cells stimulated with recombinant CD154 (Fig. 5A).

Previous work has demonstrated that the TRAF3 gene encodes different isoforms [32]. The identity of the two immunoreactive bands detected by the anti-TRAF3 polyclonal antibody in Figure 5A was verified by RNAi. It was found that transfection of EJ cells with TRAF3 siRNAs diminished the expression of the higher molecular weight and reduced the intensity of the low molecular weight protein whereas siRNA targeting the RIP1 kinase or an unrelated siRNA targeting *luciferase* had no effect (Fig. 5B). In agreement with the established link between TRAF3 and non-canonical NF-κB signaling [16], [17], the attenuated levels of TRAF3 in Ubc9-ablated EJ cells correlated with reduced p100 NF-κB2 processing upon CD40 stimulation (Fig. 5C). In contrast, activation of the canonical NF-κB pathway, as determined by the extent of nuclear translocation of the p65/RelA NF-κB subunit following CD40 activation was similar between Ubc9 and control siRNA-transfected cells (Fig. 5D).

### SUMOylation increases the CD40-interacting capacity of TRAF3

**Figure 6:**
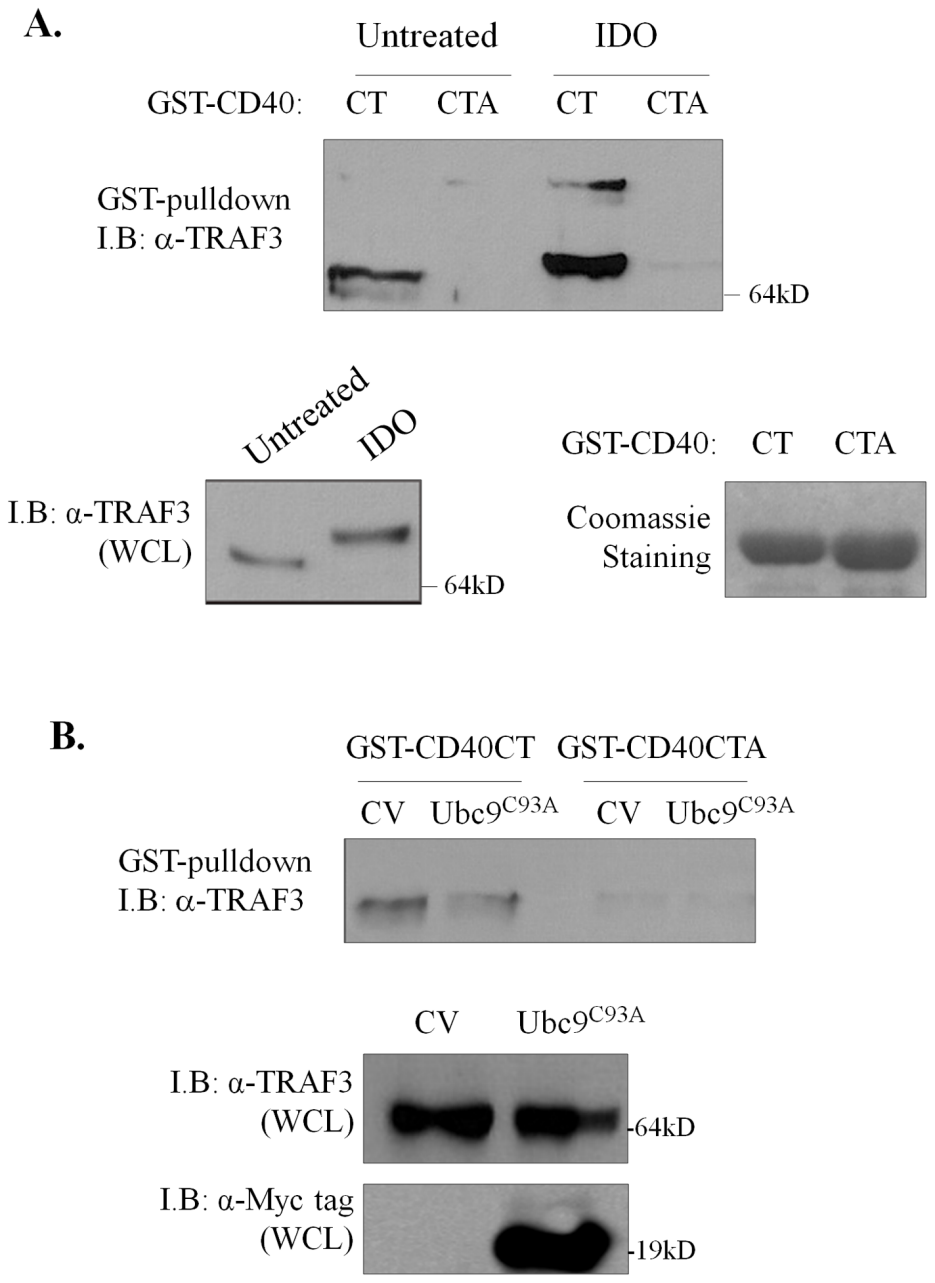
TRAF3 SUMOylation affects its CD40-interacting capacity. (**A**) The binding of TRAF3 to GST-CD40CT increases when SUMO modification is maintained. HEK293 cells were lysed in the presence or absence of iodoacetamide (IDO) and lysates were incubated with bacterially produced GST-CD40 C-terminus (CT) or, as control GST-CD40CTA, carrying a T^254^→A mutation (GST-CD40CTA) which abolishes interaction with TRAF3, bound to glutathione sepharose beads. Interacting proteins were fractionated by SDS-PAGE and immunoblotted (*I.B*.) with anti-TRAF3 Ab. Whole cell lysates (*WCL*; 30 µg) were analyzed by immunoblot for TRAF3 expression levels. Lower right panel: Coomassie-stained gel showing GST-CD40CT and GST-CD40CTA produced in bacteria. (**B**) Over-expression of “dominant-negative” Ubc9^C93A^ reduces binding of TRAF3 to CD40. HEK293 cells were transfected with Ubc9^C93A^ or control vector (*CV*), lysates were obtained using an IDO-containing lysis buffer and incubated with GST-CD40CT or GST-CD40CTA bound to glutathione sepharose beads. Interacting proteins were fractionated by SDS-PAGE and immunoblotted (*I.B*.) with anti-TRAF3. Whole cell lysates (*WCL*; 30 µg) were analyzed for TRAF3 and Ubc9^C93A^ expression levels by immunoblotting using anti-TRAF3 and Myc tag Abs, respectively. Results in (A) & (B) are representative of 3 independent experiments.

SUMOylation may influence the ability of a modified substrate to interact with partner proteins. Given that CD40 ligation does not effectively induce TRAF3 degradation following Ubc9 knock-down, we surmised that TRAF3 SUMOylation may affect the interaction of TRAF3 with CD40. To address this hypothesis, the CD40 cytoplasmic tail (CT, Fig. 6A) was produced as fusion with GST in bacteria and incubated with lysates from HEK293 cells in the presence or absence of iodoacetamide (see Fig. 3B). GST pull-downs demonstrated that CD40 strongly interacts with TRAF3 in the presence of IDO but association is reduced in the absence of SUMO protease inhibitor (Fig. 6A). As control, CD40 carrying a Thr^254^Ala mutation (CTA) known to abolish interactions with TRAF3 [3] failed to bind TRAF3 irrespective of IDO treatment.

To further validate this finding, a “dominant-negative” Ubc9^C93A^ was over-expressed in HEK293 cells and lysates were incubated, in the presence of IDO, with GST-CD40CT or GST-CD40CTA bound to glutathione sepharose beads. Interacting proteins were fractionated by SDS-PAGE and immunoblotted with anti-TRAF3. Compared to vector-transfected cultures, Ubc9^C93A^ reduced binding of TRAF3 to CD40.

## Discussion

TRAF3 is increasingly recognized as key mediator of inflammatory and oncogenic signal transduction. The pleiotropic functions of TRAF3 largely depend on its ubiquitination status with K^63^-mediated Ub linkages regulating the activation of IRF3 and the IFN response and degradative K^48^-mediated ubiquitination being required for CD40-induced non-canonical NF-κB2 signaling [20]. Results presented in this study demonstrate that TRAF3 is subject to an additional post-translational modification, SUMOylation, through its direct interaction with the SUMO conjugating enzyme Ubc9. In particular, we have found TRAF3 to be modified by both SUMO-1 and SUMO-2/3. SUMO-2/3 can be conjugated to target proteins in a chain-wise fashion due to internal SUMO conjugation motifs, whereas SUMO-1 lacks this ability and often acts as a chain terminator [33], [34]. It is thus likely that TRAF3 is modified by mixed SUMO-2/3 and SUMO-1 proteins with SUMO-1 “capping” and terminating SUMO-2/3 chains. The possibility that mono-SUMOylation also occurs requires evaluation particularly as TRAF3 contains numerous Lys residues which may serve as targets for SUMO modification.

A crosstalk between SUMOylation and ubiquitination has recently been suggested on the basis of co-purification of SUMO2/3 and ubiquitin conjugates and the accumulation of SUMO2/3 conjugates in cells treated with the proteasomal inhibitor MG132 [34], [35], [36], [37]. Whilst clear evidence that SUMO and ubiquitin are linked to one another in the same chain has still to be determined, the reported findings show that SUMO2/3-modified proteins may be ubiquitinated and targeted for proteasomal degradation [35]. Indeed, once poly-SUMOylated, the promyelocytic leukemia (PML) protein recruits the ubiquitin ligase RING finger protein 4 (RNF4) [38], [39] which elongates the SUMO chains by adding ubiquitin moieties to them. This, in turn, results in proteasomal degradation of PML [39]. SUMOylation has also been reported to positively modulate ubiquitination of the Williams-Beuren syndrome protein GTF2IRD1 [40]. Data shown in the present study provide additional support for the association between these post-translational modifications by demonstrating that Ubc9 knock-down significantly reduces CD40-mediated TRAF3 degradation and NF-κB2 processing. Despite the established role of ubiquitination in TRAF3 signal transduction [20], [41], the Lys residues targeted for ubiquitin conjugation remain unknown and further studies are required to elucidate whether TRAF3 SUMOylation affects proteasomal recognition and if substrates modified by the two types of conjugates can be degraded.

Protein modification by SUMOylation can have different outcomes including conformational changes and interactions with other proteins [36], [37]. Data presented in this paper show that SUMO modification of TRAF3 influences its CD40-interacting capacity. CD40 may exert opposing phenotypic effects on normal *versus* malignant cells [42]. The finding that TRAF3 is subject to SUMO modification irrespective of the transformation status of the cell (Fig. 3) indicates a broad role for TRAF3 SUMOylation in CD40-mediated NF-κB2 signal transduction.

On the basis of these observations we propose that SUMO-modified TRAF3 represents a pool of TRAF3 ‘primed’ for CD40 binding and stimulus-dependent ubiquitination and degradation. As TRAF3 is SUMOylated in the absence of stimulus (Fig. 3), we surmised that SUMOylation may also affect basal TRAF3 turnover. Indeed, semi-quantitative assessment of TRAF3 expression in EJ and HEK293 cells transfected with Ubc9 siRNA demonstrated elevated basal levels of TRAF3 (Fig. S1). Given the important functional roles attributed to TRAF3 [12]-[16], our findings may shed new light into the molecular pathways regulating TRAF3 function and signal transduction.

## Supporting Information

Figure S1 **Ubc9 regulates the basal levels of TRAF3.** EJ and HEK293 cells were transfected with Ubc9 siRNA or an unrelated siRNA targeting *luciferase* (Luc). Lysates (20 µg) were immunoblotted with anti-TRAF3, Ubc9 or β-actin antibodies, as indicated. Results are representative of 5 independent experiments. Semi-quantitation of TRAF3 expression was performed using Image J (http://rsbweb.nih.gov/ij/).
(TIF)
