# Supplementary figures and images for: SUMOylation Is Required for Optimal TRAF3 Signaling Capacity
Source: PLoS One. 2013 Nov 18;8(11):e80470. doi: 10.1371/journal.pone.0080470 (PMC3832365; doi:10.1371/journal.pone.0080470)

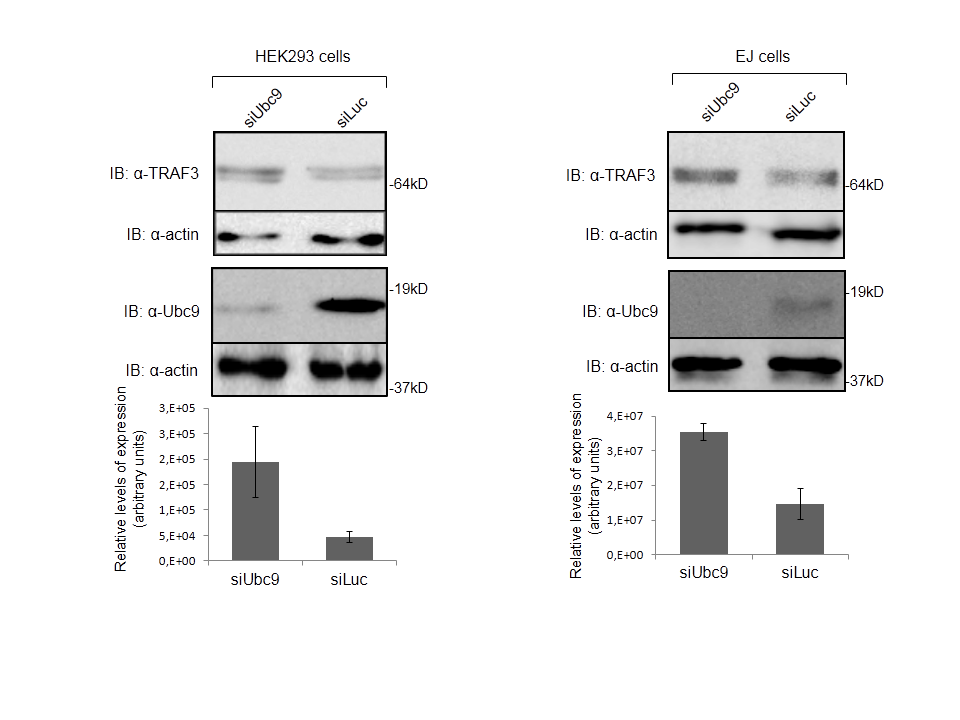

Supplement: Figure S1 — Ubc9 regulates the basal levels of TRAF3. EJ and HEK293 cells were transfected with Ubc9 siRNA or an unrelated siRNA targeting luciferase (Luc). Lysates (20 µg) were immunoblotted with anti-TRAF3, Ubc9 or β-actin antibodies, as indicated. Results are representative of 5 independent experiments. Semi-quantitation of TRAF3 expression was performed using Image J (http://rsbweb.nih.gov/ij/). (TIF) [file pone.0080470.s001.tif]
